# Utilization of a Meningitis/Encephalitis PCR panel at the University Hospital Basel – a retrospective study to develop a diagnostic decision rule

**DOI:** 10.3389/fmed.2024.1351903

**Published:** 2024-04-17

**Authors:** Andrea Erba, Fabian C. Franzeck, Vladimira Hinic, Adrian Egli, Michael Osthoff

**Affiliations:** ^1^Department of Infectious Diseases and Hospital Epidemiology, University Hospital Basel, Basel, Switzerland; ^2^Clinical Data Warehouse, Research and Analytics Services, University Hospital Basel, Basel, Switzerland; ^3^Institute of Medical Microbiology, University of Zurich, Zurich, Switzerland; ^4^Division of Clinical Bacteriology and Mycology, University Hospital Basel, Basel, Switzerland; ^5^Department of Internal Medicine, University Hospital Basel, Basel, Switzerland; ^6^Department of Internal Medicine, Cantonal Hospital Winterthur, Winterthur, Switzerland

**Keywords:** Meningitis/Encephalitis PCR panel, diagnostic stewardship, decision rule, syndromic panel, neurological infection

## Abstract

**Background:**

The Biofire^®^ FilmArray^®^ Meningitis/Encephalitis (ME) PCR panel covers 14 viral, bacterial, and fungal pathogens and has been implemented in many institutions worldwide. Post-marketing studies indicate a reduced sensitivity and overutilization underscoring the need for a more targeted usage. The aim of our study is to describe the utilization of the ME panel and to develop a diagnostic-stewardship based decision rule.

**Materials:**

Adult patients, who underwent CSF analysis with the ME panel between August 2016 and June 2021 at the University Hospital Basel, were included. Demographic, clinical, microbiological, and laboratory data were extracted from the electronic health record. Factors associated with a positive ME panel result were identified, and a decision rule was developed to potentially optimize the diagnostic yield and reduce the number of unnecessary tests.

**Results:**

1,236 adult patients received at least one panel in the observed period, of whom 106 panels tested positive (8.6%). The most frequently observed pathogens were Varicella Zoster Virus (VZV, 27%), *Streptococcus pneumoniae* (19%), Enterovirus (16%), Herpes simplex Virus 1/2 (16%), and Human Herpesvirus 6 (HHV-6, 13%). Fever, vomiting, headache, and photophobia were more frequently present in test positive patients as were significantly higher CSF leukocytes and protein concentrations. When simulating a decision rule based on CSF leukocytes and protein concentration, only 35% of all patients would have qualified for a ME panel tests, thereby increasing the positivity rate to 22.7%. 10 of 106 positive ME panels would have been missed, only involving HHV-6 and VZV (6 and 4 cases, respectively). As these subjects were either severely immunocompromised or had clinical features of shingles we propose extending the testing algorithm by including those criteria.

**Conclusion:**

The ME panel positivity rate at our institution was similar as previously published. Our results highlight the need for diagnostic-stewardship interventions when utilizing this assay by implementing a stepwise approach based on a limited number of clinical and laboratory features. This decision rule may improve the pretest probability of a positive test result, increase the quality of test utilization, and reduce costs.

## Highlights

- The positivity rate of the Meningitis/Encephalitis PCR panel was 9% at this Swiss tertiary care hospital in line with previous reports.
- The yield and benefit was very limited in patients with a CSF leucocytes count <10×10^6^/l and a protein concentration < 1,000 mg/L.
- A testing decision rule is proposed in order to improve yield of PCR panel use, which may safely increase the appropriateness of test use.

## Introduction

Central nervous system infections, such as infectious meningitis or encephalitis, are life-threating conditions caused by various agents that may remain undiagnosed (1), as their presentation may be similar to other acute central nervous system inflammatory diseases. For this reason, diagnosis can be challenging, and appropriate treatment may be delayed. It is estimated, that no pathogen is identified in approximately one-fourth to one-half of patients with acute meningoencephalitis, increasing the risk for therapy failure as well as morbidity (2, 3) and mortality. To facilitate appropriate therapy and to avoid unnecessary antimicrobial treatment, a rapid and accurate diagnosis is essential. For this purpose and utilizing a “syndromic approach,” cerebrospinal fluid (CSF) polymerase chain reaction (PCR) panels were implemented to test a sample in a short period of time for specific predefined targets (4). An example is the Biofire^®^ FilmArray^®^ Meningitis/Encephalitis (ME) PCR panel (bioMérieux, *Marcy*-*l*’*Étoile* France), a sample-to-answer, on-demand, real-time panel PCR assay for syndromic diagnosis of infectious meningitis and encephalitis from a small volume (200 μL) of cerebrospinal fluid. The test was approved by the Food and Drugs Administration (FDA) in 2015. It can detect 14 pathogens: seven viruses [Cytomegalovirus, Enterovirus, Herpes Simplex Virus 1 (HSV-1), Herpes Simplex Virus 2 (HSV-2), Human Herpesvirus 6 (HHV-6), Human Parechovirus, and Varicella Zoster Virus (VZV)], six bacteria (*Escherichia coli K1*, *Haemophilus influenzae*, *Listeria monocytogenes*, *Neisseria meningitidis*, *Streptococcus agalactiae*, and *Streptococcus pneumoniae*), and one yeast (*Cryptococcus neoformans/gattii*). Several studies showed a decreased time to diagnosis compared to classical culture-based detection, after implementation of this tool, allowing for a rapid and more appropriate antimicrobial treatment (5, 6).

However, multiple post-marketing analyses questioned the test accuracy underlining the importance of a more cautious approach to this tool. Although a pre-FDA analysis showed a > 95% sensitivity for most targets and > 99% specificity for all of them, the positive predictive value was low for some pathogens (5). Moreover, this analysis had several important limitations, including a limited number of cases, especially of *L. monocytogenes*, *N. meningitidis, E. coli K1*, and human Parechovirus. Another post-FDA retrospective study, performed in 2018, showed a reduced positive predictive value for the evaluated targets as well as a high proportion of false-negative results for HSV-1/2 (7). These results were confirmed in 2020 when a meta-analysis showed a mean sensitivity of 90% (95% confidence interval [CI], 86 to 93%) and a mean specificity of 97% (95% CI, 94 to 99%), highlighting several possible false-positive results, especially in case of bacterial targets (8, 9).

Since implementation of this panel, its diagnostic accuracy is discussed controversially, and some diagnostic pitfalls were identified as an important issue for clinicians. Although many institutions are already using it and several studies anticipate a benefit for clinical and antimicrobial stewardship outcomes (5, 10), the risk of overutilization as well as false-positive results reported in the literature, call for a more careful and evidence-based approach to this test (8, 11). Consequently, potential false-positive results should be limited by increasing pre-test probabilities. Several studies showed that diagnostic stewardship and the development of a diagnostic decision rule with a stepwise approach may improve the appropriate use of the ME Panel (12–14).

The ME panel was implemented at our institution in 2016, but no evaluation regarding its clinical usage and impact was performed. As inappropriate use of tests increases costs and the risk of unnecessary treatment and may represent a major burden to the health care system, we aimed to evaluate the utilization and interpretation of the ME panel in our hospital and to develop a diagnostic decision rule.

## Methods

A retrospective observational investigation was conducted to evaluate the implementation of the Biofire^®^ FilmArray^®^ Meningitis/Encephalitis (ME) PCR panel (bioMérieux, *Marcy*-*l*’*Étoile* France) in routine microbiology laboratory. Utilizing data available in our institution’s clinical and microbiological databases, this analysis aimed to evaluate the ME panel’s performance as a routine diagnostic in our institution. All adult in- and outpatients (≥ 18 years old) who underwent a lumbar puncture with CSF ME panel testing at the University Hospital Basel, a 700-bed teaching hospital in north-western of Switzerland, were retrospectively evaluated. The inclusion period started after the implementation of the ME panel at our institution in August 2016 until June 2021. The panel is offered across all departments since its implementation, and the analyses were conducted by the microbiological laboratory of the hospital upon request by the treating team. We excluded patients without sufficient clinical and laboratory records and patients with documented refusal regarding the use of personal or clinical data for research purposes. Patients with more than one ME panel during the same admission or outpatient care period were excluded. The study was approved by the Ethics Committee North-West and Central Switzerland (Project ID 2021-00201) with a waiver for informed consent.

Demographic, clinical, microbiological, and laboratory data were extracted from the electronic health record. The dataset included results of the ME panel as well as results from the cerebrospinal fluid routine cultures ordered by the attending physician from a single CSF sample. Laboratory analyses from cerebrospinal fluid and blood samples (inflammatory parameters, cells count, proteins, blood cultures) from the same day and the first electronically reported clinical parameters (blood pressure, respiratory rate, temperature, heart rate) were also collected. Moreover, history and clinical examination were electronically searched for key words matching with meningitis/encephalitis symptoms or clinical signs. Finally, the definitive diagnosis as mentioned in the discharge summary or consultation notes and, if applicable, infectious disease consultations regarding the ME panel result were identified Supplementary Figure S1.

Positivity rate (count of subjects with positive results divided by count of subjects with a ME panel performed) and distribution of the positive results were calculated. Clinical, laboratory and microbiological results were compared between positive and negative ME panels using appropriate statistical tests (Chi-squared test and Mann–Whitney U test, respectively) to identify factors associated with a positive result. Moreover, a suitable testing algorithm, focusing on limited clinical and laboratory parameters, was developed according to previously published articles to optimize the diagnostic yield and reduce the number of unnecessary tests. Statistical analyses were performed with SPSS statistics for Windows, version 28.0 (IBM, Armonk, NY, United States)

## Results

1,236 patients received one ME panel during the observed period, of whom 659/1236 (53.3%) were male. Median age was 59 years (IQR 42–72) and the majority of patients were hospitalized (93.1%). Most of the ME panels were ordered in the emergency department (438/1236, 35.4%) followed by the internal medicine (255/1236, 20.6%) and the neurology department (181/1236, 14.6%). The patients from emergency department showed the highest positivity rate (64/438, 14.6%), followed by the intermediate and intensive care unit (11/150, 7.3%) and the internal medicine (14/255, 5.5%). In 188/1236 (15.2%) cases the final diagnosis was a central nervous system infection. Conversely, in 97/1236 (7.8%) cases a final diagnosis was not discussed in the discharge summary.

Of 1,151 hospitalized patients, who received a ME panel, 192 (17%) patients had probable healthcare-associated meningitis (defined by ME panel prescription >3 days after admission).

Of the 1,236 performed ME panels, 106 yielded a positive result (8.6, 95% CI 7.1–10.3%). The most frequently observed pathogens were VZV (29/106, 27.4%), *Streptococcus pneumoniae* (20/106, 18.9%), Enterovirus (17/106, 16%), HSV 1/2 (17/106, 16%), and HHV-6 (14/106, 13.2%) (Supplementary Table S1). All (29/29) cases with VZV detection as well as 10/17 cases with HSV detection were due to a virus reactivation. In the remaining HSV positive cases (7/17), serological tests were not performed. Among the 14 HHV-6 positive cases, 6 were rated as relevant by the treating team and the involved infectious diseases physicians, resulting in a treatment with ganciclovir. In 7 cases, pathogen detection was considered as an innocent bystander.

In 2 instances, a singleplex HHV-6 PCR was conducted after a positive ME panel result. The first case was confirmed as positive. In the second case, the HHV-6 single PCR was negative, leading to the classification of the positive ME panel result as a false positive result.

A bacterial and fungal culture from CSF was performed in all positive tested samples. In 30/106 (28.3%) cases positive in the ME panel, microbiological growth was detected. In 12/30 (40%) positive bacterial cultures, the identified pathogen did not match the pathogen detected in the ME panel; however, all such instances were interpreted as sample contamination (e.g., identification of *Staphylococcus epidermidis*, *Cutibacterium acnes*, *Staphylococcus haemolyticus*) of which only 1 sample was drawn from an external ventricular drain. The other 18/30 (60%) positive bacterial cultures were congruent with the ME panel. An infectious disease specialist was involved in the majority of the test positive cases (79/106, 74.5%). Table 1 shows the detailed description of the clinical and laboratory results. Comparing the results between patients with positive and negative ME panels (Table 2), a significantly higher frequency of fever, vomiting, headache, and photophobia was observed in patients with a positive ME panel. Additionally, CSF leukocytes and protein concentration were significantly higher (*p*-value <0.001).

**Table 1:** Patients’ characteristics.

**Sex (*n* = 1,236)**	
Female	577 (46.7%)
Age (years) (*n* = 1,236)	59 (42–72)
Charlson comorbidity index	1 (0–2)
**Inpatient (*n* = 1,236)**	
Yes	1,151 (93.1%)
**Prescribing unit (*n* = 1,236)**	
Emergency department	438 (35.4%)
Medicine	255 (20.6%)
Neurology	181 (14.6%)
IMC/ICU	150 (12.1%)
Neurosurgery	14 (1.1%)
Surgery	8 (0.6%)
Other	190 (15.4%)
**Symptoms at presentation (*n* = 1,187)**	
Headache	420 (34.0%)
Fever	284 (23.0%)
Vomitus	159 (12.9%)
Nausea	156 (12.6%)
Photophobia	29 (2.4%)
Altered state of consciousness	24 (1.9%)
**Inflammatory markers at presentation**	
Leucocytes count in blood (G/l, *n* = 933)	8.3 (6.0–11.2)
C-reactive protein (mg/l, *n* = 1,172)	7.9 (1.7–47.9)
Diagnosis (*n* = 1,236)*	
Infectious meningitis	69 (5.6%)
Infectious encephalitis	48 (3.9%)
Infectious meningoencephalitis	34 (2.8%)
Autoimmune encephalitis	13 (1.1%)
Other cerebral infection	32 (2.6%)
Spinal or paraspinal infection	5 (0.4%)
Other diagnosis	938 (75.9%)
Diagnosis unknown	97 (7.8%)
**ME panel test result**	
Positive	106 (8.6%)

IQR, interquartile range; IMC, intermediate care unit; ICU, intensive care unit; ME panel, Meningitis−/Encephalitis PCR panel. Data are presented as median (IQR) for continuous measures, and *n* (%) for categorical measures. *as mentioned in the discharge summary or consultation notes; infectious meningitis, encephalitis or meningoencephalitis includes aseptic inflammation of the brain or meninges (without documentation of a pathogen) that was most likely caused by an infectious agent (as stated in the discharge summary or consultation notes).

**Table 2:** Comparison between positive and negative ME panels.

	ME panel positive	ME panel negative	*p*-value
**Sex**	*n* = 106	*n* = 1,130	0.613
Female	47 (44.3%)	530 (46.9%)	
**Admission**	*n* = 106	*n* = 1,130	0.085
Yes	103 (97.2%)	1,048 (92.7%)	
**History**	*n* = 104	*n* = 1,083	
Fever	41 (39.4%)	243 (22.4%)	**< 0.001**
Vomitus	26 (25%)	133 (12.3%)	**< 0.001**
Nausea	20 (19.2%)	136 (12.6%)	0.054
Altered state of consciousness	2 (1.9%)	22 (2.0%)	1.0
Photophobia	7 (6.7%)	22 (2.0%)	**0.01**
Headache	55 (52.9%)	365 (33.7%)	**< 0.001**
Seizure	7 (6.7%)	85 (7.8%)	0.848
Neurologic deficit	21(20.2%)	266 (24.6%)	0.320
**Clinical signs**			
Fever (> 38°C)	18/100 (18%)	113/1001 (11.3%)	**0.048**
Hypotension (MAP <70 mmHg)	7/100 (7%)	78/1008 (7.7%)	1.0
Tachycardia (heart rate > 100 bpm)	12/101 (11.9%)	152/1010 (15.0%)	0.392
Tachypnea (resp. r > 20/min.)	31/68 (45.6%)	231/533 (43.3%)	0.725
Hypoxia (SO2 < 94%)	8/98 (8.2%)	102/948 (10.8%)	0.493
**Laboratory findings**			
Leukocyte count in blood (G/l)*	8.3 (6.0–11.1)	7.8 (6.2–13.4)	0.928
CRP in blood (mg/l)*	5.0 (1.2–64.8)	8.2 (1.7–47.3)	0.86
Leucocyte count in CSF (G/l)	167 (28–379)	2 (1–12)	**< 0.001**
Protein concentrations in CSF (mg/l)	846 (591–1917)	438 (321–695)	**< 0.001**
**Prescribing unit**	*n* = 106	*n* = 1,130	**< 0.001**
Emergency unit	64 (60.4%)	374 (33.1%)	
Medicine	14 (13.2%)	241 (21.3%)	
Neurology	7 (6.6%)	174 (15.4%)	
IMC/ICU	11 (10.4%)	139 (12.3%)	
Neurosurgery	0 (0%)	14 (1.2%)	
Surgery	0 (0%)	8 (0.7%)	
Others	10 (9.4%)	180 (15.9%)	
**Diagnosis (*n* = 1,236)**	*n* = 106	*n* = 1,130	**< 0.001**
Infectious meningitis	44 (41.5%)	25 (2.2%)	
Infectious encephalitis	27 (25.5)	21 (1.9%)	
Infectious meningoecephalitis	17 (16.0%)	17 (1.5%)	
Autoimmune encephalitis	0 (0%)	13 (1.2%)	
Other cerebral infection	0 (0%)	32 (2.8%)	
Spinal or paraspinal infection	2 (1.9%)	3 (0.3%)	
Other diagnosis	14 (13.2%)	924 (81.8%)	
Diagnosis not verified	2 (1.9%)	95 (8.4%)	
**ID specialist consultation**	*n* = 106	*n* = 1,130	
	79 (74.5%)	357 (31.6%)	**< 0.001**

ME panel, Meningitis/Encephalitis PCR panel; MAP, mean arterial pressure; SO2, oxygen saturation; CRP, C-reactive protein; CSF, cerebrospinal fluid; IQR, interquartile range; IMC, intermediate care unit; ICU, intensive care unit; ID, infectious disease. *First blood sample on admission. Data are presented as median (IQR) for continuous measures, and *n* (%) for categorical measures.

Regarding test usage, a ME panel was performed in 495/1194 (41.5%) of CSF samples with CSF leukocytes and protein concentration within normal limits. Only 4/495 (0.8%) of these samples were positive, indicating a very low pretest probability for presence of infection with a target pathogen in this subset.

**Figure 1:**
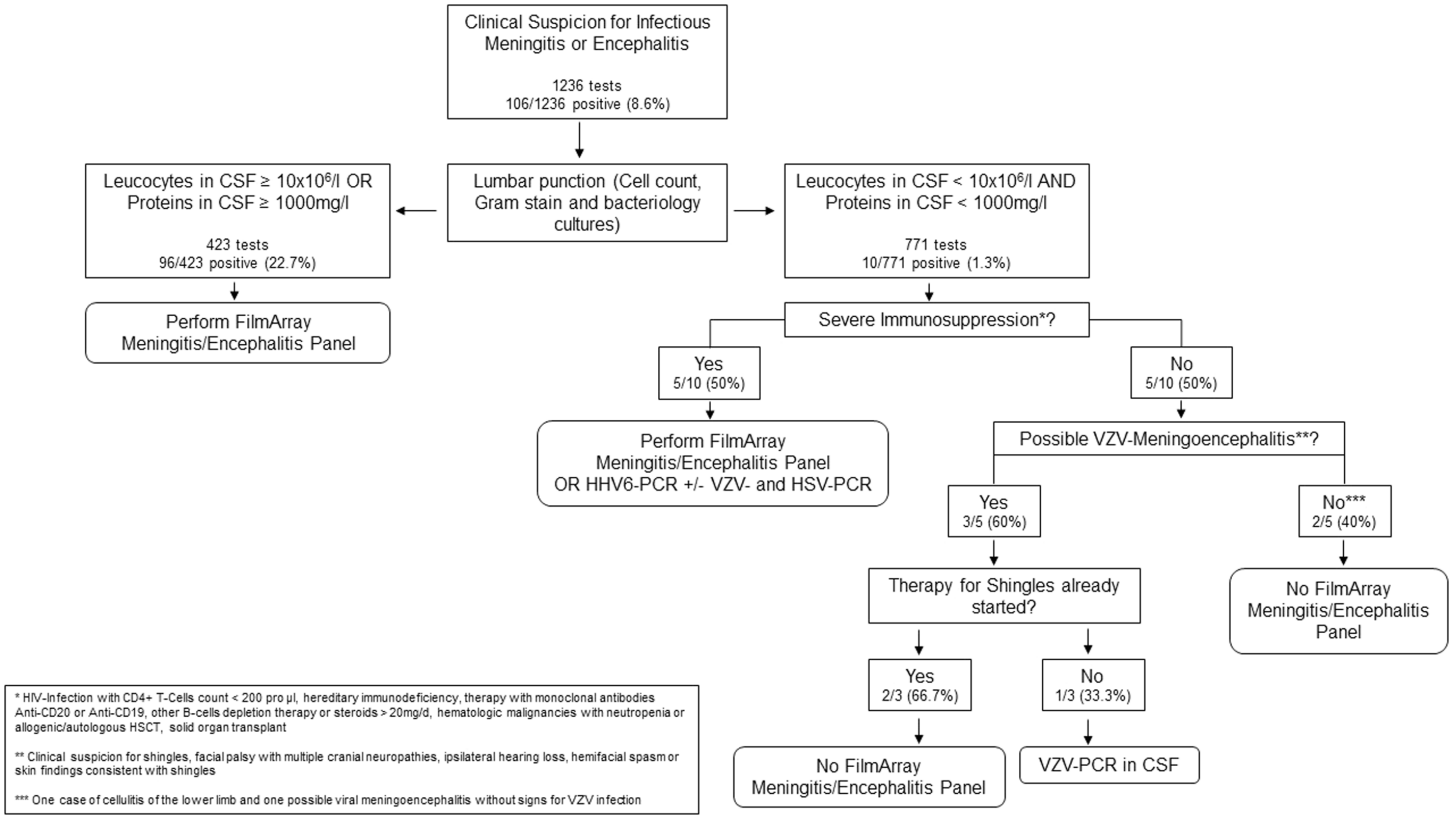
Decision rule for the use of the ME panel.

Therefore, we developed a simple decision rule based on our results and previous reports (Figure 1), recommending ordering a ME Panel conditional on results of CSF leukocyte count and CSF protein concentration. The rule would recommend performing a ME Panel on a sample if either CSF leukocyte count is ≥10 × 10^6^/l or CSF protein concentration ≥ 1,000 mg/L. Applying this rule on our study population, 423/1194 (35%) of all patients would have qualified for a ME panel, thereby increasing the positivity rate to 96/423 (22.7%). However, positive results from 10/106 patients with a positive ME panel would have been missed. Applying the decision rule on our sample would result in a sensitivity of 90.6% (95% CI 83.3–95.4%) for detecting any pathogen, when compared to testing all samples. 6/10 involved a positive result for HHV-6 of which only three were detected in immunosuppressed patients with a consecutive indication for a therapy with ganciclovir. The other three cases were rated as irrelevant by the treating team and the involved infectious diseases physicians. The other missed positive panels involved patients with VZV detection in the CSF, all of which showed clinical features of shingles. Table 3 represents the detailed characteristics of these patients. Since all the relevant missed cases involved either immunosuppression or VZV reactivation, we adapted the decision rule by incorporating criteria for HHV-6 and VZV singleplex testing (Figure 1).

**Table 3:** Detailed characteristics of patients with a positive ME panel despite a CSF leukocyte count <10 × 10^6^/l and a protein concentration < 1,000 mg/L.

Patient	Diagnosis	Panel result	Immunosuppression	Clinical presentation	Antiviral treatment
47y, male	HHV-6 encephalitis	HHV-6	Allogenic HSCT	Fever, confusion	Yes, ganciclovir
72y, male	HHV-6 encephalitis	HHV-6	Possible transient immunosuppression with low CD4 cells count, etiology unknown.	Epileptic seizure	Yes, ganciclovir
82y, female	VZV encephalitis	VZV	Yes, prednisone (20 mg/d)	Status epilepticus, shingles with involvement of thoracic dermatomes	Yes, aciclovir*
72y, male	HHV-6 encephalitis	HHV-6	Possible transient immunosuppression with low CD4 cells count, etiology unknown.	Confusion	Yes, ganciclovir
62y, male	Cellulitis lower limb	HHV-6	No	Initial presentation with Fever and headache	No
53y, female	Viral Meningoencephalitis	HHV-6	No	Fever, headache	No
73y, female	Shingles with involvement of dermatome V1	HHV-6	No	Facial palsy, shingles	Yes, aciclovir*
97y, female	Shingles V1 and VZV meningoencephalitis	VZV	No	Shingles, confusion	Yes, aciclovir*
47y, male	Ramsay hunt syndrome	VZV	Yes, prednisone (40 mg/d)	Facial palsy, ear rash	Yes, valaciclovir
63y, female	VZV associated facial palsy	VZV	No	Facial palsy	Yes, aciclovir*

HHV-6, human herpes virus 6; HSCT, hematopoietic stem cells transplantation; VZV, varicella zoster virus; *Aciclovir in increased dosage for central nervous system infection.

## Discussion

Rapid and accurate diagnostic tests for patients with a clinical suspicion for a central nervous system infection is paramount to provide immediate, appropriate, and safe treatment. Among multiple diagnostic methods, the “syndromic” PCR detection of pathogen in CSF combines reduced turn-around times, technical requirements with acceptable sensitivity and specificity (5, 7). In addition, some studies (15, 16) suggest an impact on length of stay and duration of empiric anti-infective treatment, especially in cases involving viral pathogens, not detectable by other diagnostic methods such as blood or CSF cultures. Furthermore, the high specificity of the ME panel and detection of not-treatable pathogens may promote the early cessation of unnecessary treatment (17). Nevertheless, there are several challenges regarding accuracy as well as the optimal utilization of this test, that question its use in the routine clinical setting (12, 18). At the present institution, neither the clinical utilization and usefulness of the ME panel has been evaluated nor have best practice recommendations (diagnostic stewardship) been established beforehand. Our results mirror previously reported data (11, 14) with a positivity rate of 8.6%.

Nevertheless, several studies and case reports indicate that the widespread use of this tool may generate relevant issues including delayed or wrong diagnosis and treatment due to false positive results, increased uncertainty by discordant results and increased costs (12, 19, 20). These observations warrant a more rational use of this panel to reduce unnecessary tests and costs. Development of management and testing guidelines (21, 22) and education of clinicians are essential, but, as described in previous studies (23, 24), not sufficient to reach this goal. Therefore, the use of clinical decision support may represent a good option to improve testing behavior, identifying the patient population that may benefit the most from this test and avoiding unnecessary investigations (24). In our analysis, we confirmed a widespread, indiscriminate use of the ME panel probably as a consequence of a significant overestimation of an infectious etiology underlying the presentation of the respective patients, which may be identified by the ME panel. This is underscored by the high frequency of performed panels in patients with CSF fluid cell count and protein concentration within the normal range (over 40% of patients) and the wide range and high frequency of final non-infectious diagnoses (over 70%).

For these reasons, we developed a simple decision rule based on leukocytes and protein concentration in CSF to promote a rational use of the ME panel and provide clinical decision support in patients with suspected CNS infection. These criteria were based on previous studies using similar strategies to safely reduce the ME panel use or CSF herpes virus testing (23–25). In these studies, the authors showed a safe reduction in herpes virus testing using a cutoff of 10 × 10^6^ CSF leukocytes pro liter in non-immunocompromised patients (23, 25). Similarly, McCreery et al. showed an effective and safe reduction of the ME panel use in non-immunocompromised adults with CSF leukocytes count <10 ×10^6^/l (24). Due to the reported (26) possibility of bacterial meningitis with normal cerebrospinal fluid leukocytes counts and presence of severely immunocompromised patients in our analysis, protein concentration in cerebrospinal fluid was included in our criteria. When applying this rule in our population we found that not a single bacterial infection would have been missed. 10 results positive for viruses would not have been detected with these criteria, of which 7 were clinically relevant. These results are consistent with the assumption, that viral infections can lead to a modest change in cerebrospinal fluid parameters (18, 27) and underline the important role of VZV and HHV-6 infections, especially in immunocompromised patients. Patients with cancer or immunocompromising diseases feature different characteristics, that should be taken into consideration (28). HHV-6 detection poses a relevant challenge regarding its clinical significance. According to Radmard et al. (11), who analyzed the ME panel accuracy, 10/13 positive results for HHV-6 were discordant or clinically irrelevant. Therefore, a search for this pathogen should be performed only in immunocompromised patients, with signs of meningoencephalitis.

VZV detection represents another challenge in the clinics. It was shown that shingles without neurological involvement may cause pleocytosis as well as VZV-DNA detection in CSF (29–31). Consequently, a positive result for VZV in the ME panel should be identified as relevant only in presence of a typical clinical manifestation of meningoencephalitis. This situation would be the only indication for modifying antiviral therapy (intravenous instead of oral) for VZV infection (31).

Considering these results, we decided to adapt and represent the decision rule in a flow chart to simplify the process behind ME panel use. As part of the evaluation, we included the immunological state and clinical signs for VZV meningoencephalitis. If one of these criteria is met, we advise for additional test, which may include a specific single-plex PCR or the ME panel. Using this decision rule, unnecessary tests and consequently overutilization and overreliance of the ME panel for diagnostic purposes may be avoided. Nevertheless, estimating the pretest probability of a CNS infection remains paramount before deciding to use the decision rule.

Several limitations are present in this study, including the retrospective design, with results from a single center and without specific singleplex PCRs as gold standard. However, analyzing the accuracy of the ME panel was not the intention of the present work. Another limitation is the lack of the exact number of immunocompromised patients included in the analysis, as these results could have provided a better understanding of the ME panel use in these categories of patients. Moreover, since we have not assessed the proportion of patient with a potential central nervous system infection who underwent lumbar puncture (and may have not received a ME panel), the use of the ME panel should not be based only on CSF results, but in addition to the clinician adjudicated pretest probability of a CNS infection. Furthermore, we did not analyze the indication for lumbar puncture, and data collection regarding patients’ signs and symptoms relied on a keyword search within the clinical information system. Consequently, the proportion of ME panels ordered without clinical suspicion for meningitis or encephalitis could not be evaluated, and due to lack of documentation, certain clinical events may be missing. Finally, the lack of a validation cohort warrants further studies to better evaluate the use of this decision rule.

In summary, our analysis shows that a different approach to the ME panel is needed, including a more responsible testing strategy to increase pretest probability. A decision rule, as presented in this manuscript, may safely improve the quality of test use and reduce costs.

## Data availability statement

The raw data supporting the conclusions of this article will be made available by the authors, without undue reservation.

## Ethics statement

The studies involving humans were approved by Ethics Committee North-West and Central Switzerland (Project ID 2021-00201). The studies were conducted in accordance with the local legislation and institutional requirements. The Ethics Committee/Institutional Review Board waived the requirement of written informed consent for participation from the participants or the participants’ legal guardians/next of kin because pre-existing data without correlation to the actual health situation were used.

## Author contributions

AnE: Data curation, Formal analysis, Investigation, Writing – original draft. FF: Data curation, Software, Writing – review & editing. VH: Data curation, Writing – review & editing. AdE: Data curation, Writing – review & editing. MO: Conceptualization, Data curation, Formal analysis, Investigation, Methodology, Project administration, Resources, Supervision, Writing – review & editing.
