# Supplementary material for: Utilization of a Meningitis/Encephalitis PCR panel at the University Hospital Basel – a retrospective study to develop a diagnostic decision rule
Source: Front Med (Lausanne). 2024 Apr 17;11:1351903. doi: 10.3389/fmed.2024.1351903 (PMC11061443; doi:10.3389/fmed.2024.1351903)
Supplement: Supplementary file 1 [file Data_Sheet_1.DOCX]

**Supplementary Material**

**Figure S1** Process from data collection to statistical analysis.


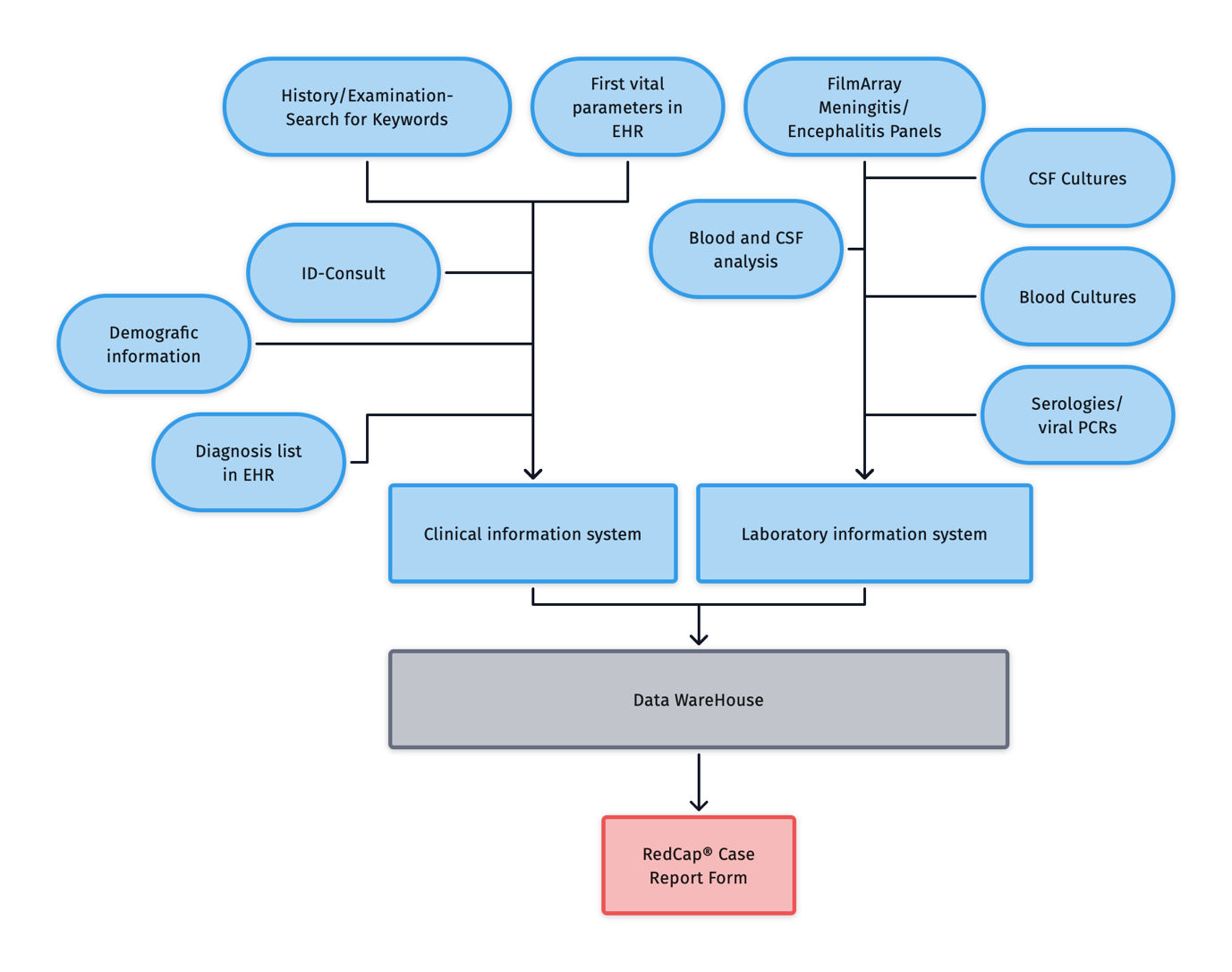


Abbreviations: ID, infectious disease; EHR, electronic health record; CSF, cerebrospinal fluid

**Table S1: Microbiological results of the cerebrospinal fluid samples tested positive with the ME panel (n=106)**

| **ME panel result: bacteria (n=27/106)** |  |
| --- | --- |
| *Escherichia coli* K1 | 1 (0.9%) |
| *Haemophilus influenzae* | 2 (1.8%) |
| *Listeria monocytogenes* | 0 (0.0%) |
| *Neisseria meningitidis* | 3 (2.8%) |
| *Streptococcus agalactiae* | 1 (0.9%) |
| *Streptococcus pneumoniae* | 20 (18.9%) |
| **ME panel result: viruses (n=78/106)** |  |
| VZV | 29 (27.4%) |
| HSV-1 | 7 (6.6%) |
| HSV-2 | 10 (9.4%) |
| Human Parechovirus | 0 (0.0%) |
| HHV-6 | 14 (13.2%) |
| Enterovirus | 17 (16.0%) |
| CMV | 1 (0.9%) |
| **ME panel result: fungi (n=1/106)** |  |
| *Cryptococcus neoformans/gattii* | 1 (0.9%) |
| **Culture from the same CSF (if ME panel positive)** |  |
| Yes | 30 (28.3%) |
| Coagulase-negative staphylococcior *Cutibacterium acnes*  *Escherichia coli*  *Neisseria meningitidis*  *Streptococcus pneumoniae*  *Cryptococcus neoformans* | 12/30 (40%)  1/30 (0.3%)  2/30 (0.7%)  14/30 (46.7%)  1/30 (0.3%) |

Abbreviations: ME panel, meningitis/encephalitis PCR panel; VZV, varicella zoster virus; HSV, herpes simplex virus; HHV-6, human herpes virus 6; CMV, cytomegalovirus
